# Evaluation of an integrated care program for thoracic surgery in Ontario, Canada: a historical cohort study

**DOI:** 10.1186/s12913-025-13049-1

**Published:** 2025-08-07

**Authors:** Nicholas Bakewell, Catherine Liang, Tsoleen Ayanian, Sanjana Sundaram, Kazuhiro Yasufuku, Meghan O’Neill, Kathy Kornas, Lori Diemert, Megan Samantha Lowe, Melissa Chang, Laura C. Rosella

**Affiliations:** 1 Dalla Lana School of Public Health, University of Toronto, 155 College St. Health Sciences Building, 6th Floor, Toronto, ON Canada; 2 University Health Network, Toronto, ON Canada; 3 Temerty Faculty of Medicine, University of Toronto, Toronto, ON Canada

**Keywords:** Integrated care, Postoperative period, Thoracic surgery, Cohort study

## Abstract

**Introduction:**

Integrated care (IC) may help to improve postoperative health outcomes among thoracic surgery patients. We conducted an outcome evaluation of an IC program implemented within the Division of Thoracic Surgery of a large hospital network in Ontario, Canada.

**Methods:**

Historical cohort design using data on patients who underwent thoracic surgery to compare outcomes of two IC groups (Pre-COVID IC: June 2019-February 2020; COVID IC: March 2020-September 2022), to a Historical non-IC group (June 2018-February 2019). Stratified by care path (low [minimally invasive procedures], medium [other procedures], high [complex procedures]), we compared risks of readmissions and emergency department (ED) visits, and mean length of stay (LOS) and healthcare costs up to 90 days post-discharge using modified Poisson/Ordinary Least Squares, Negative Binomial and Gamma regression, respectively, adjusting for age, sex, and location of residence.

**Results:**

In total, 1572 patients were included (Pre-COVID IC (*n* = 269); COVID IC (*n* = 869); Historical non-IC (*n* = 434)), with the largest proportion enrolled in the low care path. Compared to the Historical non-IC group, both the Pre-COVID and COVID IC groups had a statistically significantly shorter mean index LOS in the low (relative mean difference (RMD): 0.74 (95% confidence interval: 0.63–0.88) [Pre-COVID]; 0.66 (0.58–0.75) [COVID]) and medium (RMD: 0.75 (0.59–0.97) [Pre-COVID]; 0.62 (0.51–0.74) [COVID]) care paths; results were similar for total LOS (including readmissions). In contrast, the Pre-COVID IC group had a statistically significantly higher mean index LOS in the high care path (RMD: 1.39 (1.02–1.89)). The Pre-COVID IC group had a statistically significantly lower risk of 90-day ED visits in the low care path (relative risk (RR): 0.59 (0.36–0.97)) and a statistically significantly lower mean index cost in the medium care path (RMD: 0.72 (0.52–0.99)). The COVID IC group had a statistically significantly lower risk of 90-day readmissions in the low care path (RR: 0.64 (0.42–0.97)).

**Conclusions:**

IC programs may reduce post-discharge ED visits, LOS and healthcare costs for thoracic surgery patients, particularly for those in a low care path. As the IC program continues, further research is needed with larger sample sizes to confirm these findings and optimize IC program delivery, especially for more complex surgical patients.

**Supplementary Information:**

The online version contains supplementary material available at 10.1186/s12913-025-13049-1.

## Background

In traditional care models, acute care is predominantly managed by hospital providers. Following discharge, care is commonly shifted onto external home care providers, patients, and their caregivers [1]. The post-discharge period raises challenges for patients and caregivers, including the stresses of caregiving, disruptions to continuity of care and transfers of medical information, and communication gaps between care providers [2–4], all of which can contribute to poor health outcomes, avoidable acute care visits and additional healthcare costs [5–7]. This is particularly the case for thoracic surgery patients, where postoperative care can be challenging, and patients experience high rates of postoperative morbidity and mortality [8–10].

Much evidence exists to support the effectiveness of integrated care (IC) interventions that bridge the gap during the hospital-to-community transition process in improving patient outcomes [11–13]. Established guidelines for the care of patients undergoing thoracic surgery emphasize the importance of the pre- and post-discharge periods, as well as the transitional periods between them [14, 15]. More recently, a small body of research has specifically demonstrated varying degrees of effectiveness of IC interventions in reducing hospital length of stay (LOS) and post-discharge emergency department (ED) visits in the thoracic surgery population [16–18].

In response to calls for health system transformation to improve patient outcomes undergoing acute care, the University Health Network (UHN), a major network of hospitals in Toronto, Ontario, Canada, developed the IC Program initially implemented within the Division of Thoracic Surgery. This IC Program includes sorting patients into low, medium, and high care paths, which consider factors such as the specific surgical procedure received (e.g., minimally invasive vs. more complex resections), patient comorbidities, and social determinants of health; a detailed description of the care path criteria is provided in the Methods section. The IC Program was designed according to the Quadruple Aim framework that advocates for improving patient experience, advancing population health, reducing costs, and improving provider satisfaction [19, 20]. The goals of the IC Program are to ensure integration of acute and home care; to enhance patient and caregiver experiences; to improve communications, care coordination, and understanding of the patient’s care plan across providers; and to improve the work-life balance of providers [21]. Results from the impact of the IC Program on patients, caregivers, and providers are available elsewhere [22, 23].

In this historical cohort study, we conducted an outcome evaluation of the Thoracic Surgery department’s IC Program to determine the effect on healthcare utilization and costs up to 90 days post-discharge from the index surgical visit, compared to a Historical non-IC control group. The outcomes of interest were readmissions, ED visits, LOS, and healthcare costs incurred up to 90 days post-discharge. These metrics are commonly used to assess hospital and home care quality and performance in Ontario and across Canada [24–27].

## Methods

### Study design

We conducted an outcome evaluation of the IC Program using a historical cohort design.

### Setting

The IC Program was based on the Integrated Comprehensive Care (ICC) program at St. Joseph’s Healthcare Hamilton (SJHH), with modifications for implementation within the Division of Thoracic Surgery at UHN [16, 18, 28, 29]. Specifically, the IC Program consists of a care team comprising an IC Lead who is a registered nurse serving as the primary point of contact; one shared digital record; access to a 24/7 phone line; and one integrated fund for bundled services and payments. IC Program services are delivered pre-admission, during hospitalization, and up to 90 days post-discharge from hospital, including check-in phone calls from the IC Lead, additional phone calls and home care visits by nurses, allied health team members, and personal support services depending on the patient’s care path. The IC Program was available to patients who lived in the Greater Toronto Area (GTA; includes the City of Toronto and four surrounding municipalities), and homecare was provided by Visiting Homemakers Association (VHA) Home HealthCare.

IC Program patients were sorted into surgical care paths representing care needs based on a simultaneous and holistic assessment of the procedures received, patient health status (e.g., comorbidities), potential complications, and social determinants of health (e.g., caregiver support, food insecurity, transportation). Low care path patients received minimally invasive lung and mediastinal resection (Video-assisted thoracoscopic surgery (VATS) or Robotic assisted thoracoscopic surgery (RATS)), VATS thymectomy, and pleuroscopy. High care path patients received extrapleural pneumonectomy, pulmonary endarterectomy, and esophagectomy. Patients receiving other thoracic procedures were placed in the medium care path. Patients undergoing same-day procedures were classified as an “Other” care path, distinct from the low, medium, and high care paths, due to their typically different care needs and resource utilization. These factors would indicate to the IC Lead which care path the patient would be most appropriately assigned to. Due to limitations in the historical data, care path assignment for the historical control group was based solely on the surgical procedure received. Data on comorbidities and social determinants of health were not available for these patients.

The IC Program began on June 1, 2019, with an initial roll out within the Division of Thoracic Surgery for eligible patients in the low care path and increasing enrollment over time of more complex patients in medium and high care paths.

### Data sources

Data on patient characteristics, visits associated with the initial thoracic surgical procedure (i.e., index surgical visit), and any post-index visits made within 90 days were from the Discharge Abstract Database (DAD) [30] and National Ambulatory Care Reporting System (NACRS) [31] databases; noting that DAD and NACRS data on post-index visits at non-UHN hospitals were accessed through the Ontario Hospital Association’s Integrated Decision Support (IDS) database [32]. Costs associated with visits made and procedures received were from the UHN Case Costing database. Surgical wait time data were from the Government of Ontario’s Wait Time Information System [33]. Home care utilization data were from the VHA Home HealthCare database.

The unit of analysis for all data sources was limited to data associated with the index surgical visit.

### Eligibility criteria

Enrolled IC Program patients were included in this outcome evaluation if they were sorted into a care path, had undergone thoracic surgery, and lived in the GTA (i.e., received homecare services from VHA Home HealthCare). To mitigate selection bias and given the interest is in evaluating the program among all patients enrolled, not only restricted to those alive at the end of follow-up, patients who died during follow-up were retained in analyses and may be viewed as being censored.

### Comparison groups

We identified two temporal intervention groups consisting of patients in the IC program during the Pre-COVID and COVID time periods. The Pre-COVID IC intervention group was defined as thoracic surgery patients who participated in the IC Program and were discharged from their index surgical visit from June 1, 2019 to February 29, 2020. The COVID-IC intervention group included IC Program participants who were discharged from their index surgical visit between March 1, 2020 to September 30, 2022. The COVID IC period covers from March 1, 2020, to September 30, 2022, a period encompassing the most acute phase of the COVID-19 pandemic, with the end date chosen due to complete data constraints for a comprehensive analysis. While COVID-19 impacts are ongoing, this timeframe provides valuable insights into the IC Program during a critical period. The Historical non-IC comparison group comprised historical thoracic patients from the GTA who underwent surgery and were discharged from June 1, 2018 to February 28, 2019, before the initiation of the IC Program. Historical non-IC patients were categorized into low, medium, and high care paths according to their surgical procedures for comparison purposes.

### Outcomes

Readmissions were inpatient hospitalizations, either direct entry or via the ED, that occurred after a patient was discharged from their index surgical visit. For a holistic assessment, we did not restrict our analyses to unscheduled readmissions because all readmissions come with clinical implications and add to healthcare costs. ED visits were those that occurred after the patient was discharged from their index surgical visit. Risks of readmissions and ED visits were calculated as the proportion of patients with at least one readmission and ED visit among all patients, respectively. Risks of readmissions and ED visits were calculated for 30, 60, and 90 days post-discharge.

Index LOS was the number of days upon admission to discharge for the index surgical visit. Total LOS was the number of days spent at the index surgical visit plus any readmissions up to 90 days after discharge. Visits for which admission and discharge occurred on the same day were assigned a LOS of 1 day.

Healthcare costs for the index surgical visit were the sum of dollar amounts for direct (e.g., nursing, operating room, lab, pharmacy, and allied health) and indirect overhead (e.g., administrative, information technology, and maintenance) functions. Total healthcare costs were the costs for the index surgical visit plus healthcare costs for all readmissions and ED visits up to 90 days after discharge. We excluded patients without operating room costs at their index surgical visit, as the data for these patients were considered potentially erroneous and incomplete. Refer to the Appendix for additional details on defining healthcare costs.

Surgical wait times were calculated as the number of days from first referral to first clinical appointment and the number of days from decision to surgery. Additionally, home care utilization was summarized for services provided in-person, virtually or by telephone by the UHN IC Lead, nurses, allied health professionals (i.e., registered dietician, physiotherapist, occupational therapist, social worker, speech language pathologist) and personal support workers. This analysis was restricted to the COVID-IC group because reliable home care data were only available from November 2020 onwards.

### Statistical analyses

Baseline characteristics of each group were described using means (standard deviations (SDs)), medians (interquartile ranges (IQRs) and ranges), and frequencies (percentages), as appropriate. Demographics included sex, age, and location of residence within the GTA (defined as i) residing in the City of Toronto (yes/no); and ii) mapped to their local health planning region). We examined differences in baseline characteristics between the IC groups relative to the Historical non-IC group, using a 0.10 threshold for the absolute standardized differences (ASDs) [34]. Due to the skewness of continuous variables, we used the ranks to compute the ASDs. We report one ASD for each categorical variable to assess whether there are differences in the distribution across all categories.

In statistical modelling, we stratified by care path and adjusted for age, sex, and location of residence (within the GTA; i.e., within vs. outside the Toronto Central local health planning region). To compare the absolute and relative differences in risks for readmissions and ED visits between groups, we used Poisson generalized linear models (GLMs) with a log-link and robust standard errors (i.e., modified Poisson regression) to produce risk ratios (RRs) and ordinary least squares (OLS) linear regression with robust standard errors to produce risk differences (RDs) [35, 36].

To compare index and total LOS between groups, we used negative binomial GLMs to account for overdispersion with log and identity links to produce absolute and relative adjusted differences in mean LOS, respectively [37]. To assess sensitivity of results to assuming a negative binomial distribution, we re-fitted the GLMs assuming a Poisson distribution with robust standard errors. We also conducted sensitivity analyses restricted to postoperative index LOS, which is the difference between the discharge date and surgery date. To assess sensitivity of results limited to the UHN data, we supplemented data on readmissions, ED and LOS that account for visits at non-UHN sites.

To compare index and total healthcare costs between groups, we used Gamma GLMs to account for the skewed distribution of costs with log [38] and identity links to produce absolute and relative adjusted differences in mean costs between groups, respectively. To assess sensitivity of results to assuming a Gamma distribution, we re-fitted the GLMs assuming a Gaussian distribution. We also conducted sensitivity analyses subtracting operating room costs, as these costs are beyond the control of the IC Program; and further restricting to variable direct costs, as these are costs the IC Program could impact.

As a sensitivity analysis to assess potentially insufficient adjustment of measured confounders, we re-fitted the GLMs used for the primary adjusted analyses (age, sex, location of residence) of risks for readmissions and ED visits, LOS and healthcare costs to also adjust for surgical wait time from decision to surgery. Surgical wait time from decision to surgery was included in this sensitivity analysis as a proxy to further adjust for patient severity and case complexity beyond stratification by care path. These analyses were restricted to only those with surgical wait time data. We conducted model diagnostics to evaluate model assumptions, including, for example, outliers/examined residuals, linearity of continuous predictors (i.e., age), and checking model fit statistics (e.g., Akaike information criterion).

It is important to acknowledge that the use of different link functions assume different relationships between the dependent and independent variables. The log link assumes a multiplicative relationship between an outcome and the IC program while the identity link assumes an additive relationship between the outcome and the IC program. We report results from models using these different link functions to provide a comprehensive evaluation of the IC program. While there may be instances where statistical inferences using different link functions do not coincide, it is important for the reader not to overinterpret these instances.

All analyses were performed using R version 4.2.2 and a two-sided significance level of 0.05 was used for statistical significance [39].

### Patient and public involvement

This research was undertaken with input from an Evaluation Working Group composed of patient representatives, hospital staff, and experts, who advised on the research questions, indicators, and contributed to the interpretation of findings.

## Results

### Patient characteristics

There were 1572 patients included: 269 in the Pre-COVID IC, 869 in the COVID IC, and 434 in the Historical non-IC group. There were slight differences in the sex distribution between groups, where the majority of patients in the IC groups were female (Pre-COVID IC: 54.6%, COVID IC: 52.8%), while just under half of the Historical non-IC group were female (47.7%; ASDs: 0.14, 0.10). Primary lung cancer was the most common primary diagnosis at the surgical index visit, however, there was a notably smaller proportion within the Historical non-IC group relative to the IC groups (ASDs: 0.28, 0.26). The distribution of patients across care paths showed a small imbalance between Pre-COVID IC and Historical non-IC groups (ASD: 0.11) and was notably different between the COVID IC and Historical non-IC groups (ASD: 0.29) (Table 1). In total, 5 patients died during follow-up, 2 within each of Historical Non-IC and Pre-COVID IC groups, and 1 within the COVID IC group.

**Table 1:** Baseline characteristics of integrated care (IC) and historical non-IC groups

	Pre-COVID IC (*N* = 269)		COVID IC (*N* = 869)		Historical non-IC (*N* = 434)		Absolute Standardized Difference^ꝉ^	
	Mean (SD)	Median (IQR)	Mean (SD)	Median (IQR)	Mean (SD)	Median (IQR)	Pre-COVID IC vs. Historical	COVID IC vs. Historical
Age (years)	62 (14)	65 (55, 73)	62 (15)	64 (54, 72)	61 (16)	64 (53, 71)	0.13	0.06
	N (%)		N (%)		N (%)			
Female (vs. Male)	147 (54.6%)		459 (52.8%)		207 (47.7%)		0.14	0.10
Primary Diagnosis							0.28	0.26
Primary lung cancer	132 (49.1%)		389 (44.8%)		163 (37.6%)			
Metastases of respiratory and digestive organs	31 (11.5%)		108 (12.4%)		66 (15.2%)			
Malignant neoplasm of thymus	10 (3.7%)		52 (6.0%)		15 (3.5%)			
Primary esophageal cancer	9 (3.3%)		29 (3.3%)		18 (4.1%)			
Pneumothorax and air leak	7 (2.6%)		22 (2.5%)		27 (6.2%)			
Other^*^	80 (29.7%)		269 (31.0%)		145 (33.4%)			
Care Path							0.11	0.29
Low	177 (65.8%)		520 (59.8%)		300 (69.1%)			
Medium	65 (24.2%)		243 (28.0%)		96 (22.1%)			
High	26 (9.7%)		82 (9.4%)		38 (8.8%)			
Other^**^	1 (0.4%)		24 (2.8%)		0 (0.0%)			
Month of Discharge^***^							0.46	1.01
June & July	20 (7.4%)		199 (22.9%)		93 (21.4%)			
August	26 (9.7%)		96 (11.0%)		44 (10.1%)			
September	46 (17.1%)		100 (11.5%)		39 (9.0%)			
October	34 (12.6%)		69 (7.9%)		55 (12.7%)			
November	40 (14.9%)		49 (5.6%)		53 (12.2%)			
December	38 (14.1%)		44 (5.1%)		48 (11.1%)			
January	32 (11.9%)		28 (3.2%)		50 (11.5%)			
February	33 (12.3%)		46 (5.3%)		52 (12.0%)			
March	0 (0.0%)		114 (13.1%)		0 (0.0%)			
April	0 (0.0%)		60 (6.9%)		0 (0.0%)			
May	0 (0.0%)		64 (7.4%)		0 (0.0%)			
Discharged to Home	266 (98.9%)		863 (99.3%)		421 (97.0%)		0.13	0.17
Resides in City of Toronto^****^	155 (57.6%)		530 (61.0%)		262 (60.4%)		0.06	0.01
Local Health Planning Region (Residence)							0.15	0.15
Toronto Central	96 (35.7%)		346 (39.8%)		152 (35.0%)			
Central	79 (29.4%)		244 (28.1%)		130 (30.0%)			
Central East	50 (18.6%)		122 (14.0%)		75 (17.3%)			
Central West	16 (5.9%)		68 (7.8%)		41 (9.4%)			
Mississauga Halton	28 (10.4%)		89 (10.2%)		36 (8.3%)			

*IC* Integrated Care, *SD* Standard Deviation, *IQR* Interquartile Range (Quartile 1, Quartile 3)

^ꝉ^Ranks were used for the standardized differences of continuous variables due to the skewed distributions

^*^The “Other” Primary Diagnosis includes diagnoses such as, but not limited to: *Malignant neoplasm of thymus*,* Myasthenia gravis*,* Other secondary pulmonary hypertension*,* Diaph hernia without obs or gangrene*,* Malignant neoplasm of cardia*

^**^The “Other” care path refers to patients who received a same day procedure

^***^Discharge months: June 2019-February 2020 for Pre-COVID IC group; March 2020-September 2022 for COVID IC group; and June 2018-February 2019 for Historical non-IC group

^****^Resides in Toronto defined as residential postal code beginning with “M”

### Readmissions

In the 90-day post-discharge period, the observed proportions with any readmission visit to a UHN site were 9.3%, 9.8% and 14.5% among Pre-COVID IC, COVID IC and Historical non-IC groups, respectively. Among those with readmissions, 58.1%, 70.5% and 66.7% were admitted through the ED rather than directly as an inpatient within the respective groups; the count distributions between groups were mostly comparable at 90 days post-discharge (Table 2).

**Table 2:** Readmissions and ED visits to the UHN post-discharge, observed

Days post-discharge	Pre-COVID IC				COVID IC				Historical non-IC			
	Risk		Count*		Risk		Count*		Risk		Count*	
	Denominator	n (%)	Median (IQR)	Min- Max	Denominator	n (%)	Median (IQR)	Min- Max	Denominator	n (%)	Median (IQR)	Min- Max
Readmissions												
90 days	269	25 (9.3%)	1 (1, 1)	1–2	869	85 (9.8%)	1 (1, 1)	1–3	434	63 (14.5%)	1 (1, 1)	1–3
ED^**^	31	18 (58.1%)			105	74 (70.5%)			75	50 (66.7%)		
60 days	269	21 (7.8%)	1 (1, 1)	1–2	869	66 (7.6%)	1 (1, 1)	1–3	434	44 (10.1%)	1 (1, 1)	1–3
ED^**^	24	15 (62.5%)			78	58 (74.4%)			55	44 (80.0%)		
30 days	269	15 (5.6%)	1 (1, 1)	1–2	869	51 (5.9%)	1 (1, 1)	1–2	434	29 (6.7%)	1 (1, 1)	1–3
ED^**^	16	11 (68.8%)			56	43 (76.8%)			36	33 (91.7%)		
ED Visits												
90 days	269	30 (11.2%)	1 (1, 1)	1–4	869	130 (15.0%)	1 (1, 2)	1–3	434	87 (20.0%)	1 (1, 2)	1–6
60 days	269	27 (10.0%)	1 (1, 1)	1–4	869	110 (12.7%)	1 (1, 1)	1–3	434	72 (16.6%)	1 (1, 2)	1–6
30 days	269	20 (7.4%)	1 (1, 1)	1–4	869	89 (10.2%)	1 (1, 1)	1–3	434	56 (12.9%)	1 (1, 1)	1–4

Risk defined as proportions with any readmissions (or ED visits) among all patients in each group. ED = unscheduled or urgent inpatient readmissions via the emergency department

*IC* Integrated Care, *IQR* Interquartile Range (Quartile 1, Quartile 3)

^*^The count summaries are among those with readmissions/ED visits, hence why 0 is not the minimum value

^**^The denominator for the “ED” rows for readmissions pertains to the number of readmissions, not patients

After adjustment, the estimated RR for the Pre-COVID IC group in the low care path indicated a lower, albeit not statistically significant, 90-day readmission risk relative to the Historical non-IC group in the low care path (RR: 0.66 (95% confidence interval: 0.37–1.19)). The estimated RR for the COVID IC group in the low care path indicated a statistically significantly lower 90-day readmission risk (0.64 (0.42–0.97)).

After adjustment, the estimated RR for the Pre-COVID IC group in the medium care path indicated a lower, albeit not statistically significant, 90-day readmission risk relative to the Historical non-IC group in the medium care path (0.88 (0.41–1.92)). Similarly, the estimated RR for the COVID IC group in the medium care path indicated a lower, albeit not statistically significant, 90-day readmission risk (0.62 (0.33–1.16)).

After adjustment, the estimated RR for the Pre-COVID IC group in the high care path indicated a lower, albeit not statistically significant, 90-day readmission risk relative to the Historical non-IC group in the high care path (0.15 (0.02–1.15)). Similarly, the estimated RR for the COVID IC group in the high care path indicated a lower, albeit not statistically significant, 90-day readmission risk (0.75 (0.36–1.56)) (Table 3).

**Table 3:** Readmission and ED visit risks, adjusted differences between IC and historical groups

Days post-discharge	Care Path	Relative Risk		(Absolute) Risk Difference	
		Pre-COVID IC (95% CI)	COVID IC (95% CI)	Pre-COVID IC (95% CI)	COVID IC (95% CI)
Readmissions					
90 days	Low	0.66 (0.37, 1.19)	0.64 (0.42, 0.97)	−4.27 (−10.03, 1.49)	−4.60 (−9.09, −0.12)
	Medium	0.88 (0.41, 1.92)	0.62 (0.33, 1.16)	−1.82 (−13.05, 9.42)	−5.91 (−14.44, 2.63)
	High	0.15 (0.02, 1.15)	0.75 (0.36, 1.56)	−22.48 (−39.01, −5.94)	−6.60 (−23.83, 10.64)
60 days	Low	0.80 (0.41, 1.53)	0.61 (0.37, 1.00)	−1.90 (−7.11, 3.32)	−3.67 (−7.56, 0.21)
	Medium	1.16 (0.45, 2.98)	0.84 (0.38, 1.84)	1.44 (−8.17, 11.04)	−1.41 (−8.44, 5.62)
	High	0.20 (0.02, 1.64)	1.02 (0.44, 2.36)	−15.11 (−29.91, −0.32)	0.27 (−15.10, 15.63)
30 days	Low	0.82 (0.37, 1.82)	0.71 (0.39, 1.30)	−1.07 (−5.38, 3.23)	−1.76 (−4.99, 1.47)
	Medium	1.07 (0.36, 3.16)	0.79 (0.31, 1.99)	0.45 (−7.77, 8.67)	−1.49 (−7.87, 4.89)
	High	0.35 (0.04, 3.14)	1.57 (0.54, 4.61)	−7.09 (−19.48, 5.30)	6.01 (−6.76, 18.79)
ED Visits					
90 days	Low	0.59 (0.36, 0.97)	0.73 (0.53, 1.01)	−7.47 (−13.93, −1.00)	−4.87 (−10.13, 0.39)
	Medium	0.50 (0.22, 1.10)	0.62 (0.38, 1.01)	−11.03 (−22.11, 0.05)	−8.29 (−17.67, 1.08)
	High	0.49 (0.17, 1.39)	0.84 (0.45, 1.56)	−16.30 (−37.30, 4.70)	−5.02 (−23.48, 13.43)
60 days	Low	0.66 (0.39, 1.11)	0.73 (0.51, 1.04)	−5.25 (−11.47, 0.96)	−4.20 (−9.15, 0.74)
	Medium	0.54 (0.23, 1.30)	0.69 (0.40, 1.21)	−7.59 (−17.58, 2.39)	−4.78 (−13.20, 3.63)
	High	0.42 (0.12, 1.43)	0.88 (0.44, 1.76)	−15.59 (−34.72, 3.54)	−3.19 (−20.77, 14.40)
30 days	Low	0.57 (0.31, 1.04)	0.73 (0.49, 1.08)	−5.57 (−11.07, −0.07)	−3.48 (−8.02, 1.06)
	Medium	0.73 (0.27, 1.97)	0.79 (0.38, 1.63)	−2.80 (−11.36, 5.77)	−2.02 (−9.23, 5.19)
	High	0.36 (0.08, 1.64)	0.94 (0.43, 2.09)	−13.46 (−30.48, 3.56)	−1.20 (−17.53, 15.14)

Adjusted for age, sex, and local health planning region (within vs. outside the Toronto Central local health planning region), using the Historical non-IC group as the reference group

Sample sizes of each care path: low (*N* = 997), medium (*N* = 404), high (*N* = 146)

*IC* Integrated Care, *95% CI* 95% confidence interval

### ED visits

In the 90-day post-discharge period, the observed proportions with any ED visit to a UHN site were 11.2%, 15.0% and 20.0% among Pre-COVID IC, COVID IC and Historical non-IC groups, respectively. Among those with ED visits, the count distributions between groups differed at 90 days post-discharge, with the Historical non-IC group having a higher maximum count of ED visits of 6 relative to a maximum of 4 and 3 for the Pre-COVID and COVID IC groups, respectively (Table 2).

After adjustment, the estimated RR for the Pre-COVID IC group in the low care path indicated a statistically significantly lower 90-day ED visit risk relative to the Historical non-IC group in the low care path (0.59 (0.36–0.97)). The estimated RR for the COVID IC group in the low care path indicated a lower, albeit not statistically significant, 90-day ED visit risk (0.73 (0.53–1.01)).

After adjustment, the estimated RR for the Pre-COVID IC group in the medium care path indicated a lower, albeit not statistically significant, 90-day ED visit risk relative to the Historical non-IC group in the medium care path (0.50 (0.22–1.10)). Similarly, the estimated RR for the COVID IC group in the medium care path indicated a lower, albeit not statistically significant, 90-day ED visit risk (0.62 (0.38–1.01)).

After adjustment, the estimated RR for the Pre-COVID IC group in the high care path indicated a lower, albeit not statistically significant, 90-day ED visit risk relative to the Historical non-IC group in the high care path (0.49 (0.17–1.39)). Similarly, the estimated RR for the COVID IC group in the high care path indicated a lower, albeit not statistically significant, 90-day ED visit risk (0.84 (0.45–1.56)) (Table 3).

### Length of stay

The observed median index and total LOS were 3 days across all groups. However, the Historical non-IC group tended to have greater variation for both index and total LOS relative to the IC groups, as indicated by the larger SD and range (TableA4).

After adjustment, the estimated relative mean difference (RMD) for the Pre-COVID IC group in the low care path indicated a statistically significantly shorter mean index LOS relative to the Historical non-IC group in the low care path (RMD: 0.74 (0.63–0.88)). Similarly, the estimated RMD for the COVID IC group in the low care path indicated a statistically significantly shorter mean index LOS relative (0.66 (0.58–0.75)).

After adjustment, the estimated RMD for the Pre-COVID IC group in the medium care path indicated a statistically significantly shorter mean index LOS relative to the Historical non-IC group in the medium care path (0.75 (0.59–0.97)). Similarly, the estimated RMD for the COVID IC group in the medium care path indicated a statistically significantly shorter mean index LOS relative (0.62 (0.51–0.74)).

In contrast, after adjustment, the estimated RMD for the Pre-COVID IC group in the high care path indicated a statistically significantly longer mean index LOS relative to the Historical non-IC group in the high care path (1.39 (1.02–1.89)). Similarly, the estimated RMD for the COVID IC group in the high care path indicated a longer, albeit not statistically significantly, mean index LOS (1.13 (0.89–1.44)) (Table 4).

**Table 4:** Adjusted differences in length of stay and case costing outcomes for integrated care (IC) vs. historical non-IC patient groups (Negative binomial (Type 2) GLM for length of stay outcomes and gamma GLM for case costing outcomes)

	Care Path	Relative Difference (95% CI)		Absolute Difference (95% CI)^*^	
		Pre-COVID IC	COVID IC	Pre-COVID IC	COVID IC
Length of Stay (LOS) Outcomes					
Index Hospitalization LOS	Low	0.74 (0.63, 0.88)	0.66 (0.58, 0.75)	−1.17 (−1.79, −0.55)	−1.51 (−2.01, −1.02)
	Medium	0.75 (0.59, 0.97)	0.62 (0.51, 0.74)	−2.04 (−3.80, −0.27)	−3.17 (−4.56, −1.77)
	High	1.39 (1.02, 1.89)	1.13 (0.89, 1.44)	4.04 (−0.12, 8.20)	1.53 (−1.25, 4.31)
Total LOS (Index + Readmissions)	Low	0.73 (0.61, 0.87)	0.67 (0.59, 0.77)	−1.48 (−2.24, −0.71)	−1.80 (−2.42, −1.19)
	Medium	0.73 (0.55, 0.96)	0.64 (0.52, 0.79)	−2.50 (−4.74, −0.25)	−3.36 (−5.16, −1.56)
	High	1.14 (0.84, 1.57)	1.02 (0.80, 1.30)	1.70 (−2.89, 6.30)	0.34 (−3.00, 3.68)
Case Costing Outcomes					
Index Visit Costs	Low	0.76 (0.58, 1.00)	0.96 (0.77, 1.20)	−3670.17 (−7214.66, −125.69)	−572.00 (−3849.83, 2705.83)
	Medium	0.72 (0.52, 0.99)	0.80 (0.62, 1.03)	−6700.31 (−13360.12, −40.49)	−4730.36 (−10515.43, 1054.70)
	High	1.23 (0.80, 1.90)	1.38 (0.97, 1.95)	5914.97 (−11123.43, 22953.36)	12878.43 (−1753.28, 27510.13)
Total Costs (Index + Post-discharge costs)	Low	0.79 (0.60, 1.04)	0.99 (0.80, 1.24)	−3386.09 (−7148.68, 376.50)	−3.48 (−3474.55, 3467.60)
	Medium	0.69 (0.47, 1.00)	0.79 (0.59, 1.07)	−8166.50 (−16757.41, 424.40)	−5343.73 (−12906.41, 2218.95)
	High	1.13 (0.74, 1.72)	1.32 (0.94, 1.86)	2618.42 (−14558.66, 19795.51)	12213.52 (−3096.12, 27523.15)

*IC* Integrated Care, *95% CI* 95% confidence interval

Reference group = historical non-IC group

All LOS and case costing estimates presented were adjusted for age, sex, and local health planning region (within vs. outside the Toronto Central local health planning region)

^*^Absolute difference effect estimates are in days for length of stay outcomes, and Canadian dollars ($) for cost outcomes

The results observed for total LOS in the low, medium and high care paths was similar in the IC groups, compared to the historical non-IC group (Table 4).

### Healthcare costs

The median cost of the index surgical visitwas (Canadian dollars (CAD$)) $11,472, $14,285 and $10,903 for the Pre-COVID IC, COVID IC and Historical non-IC groups, respectively. Among those with a readmission and/or ED visit 90 days post-discharge, the median cost for readmission and/or ED visits was $3881, $4028 and $2437 for the Pre-COVID IC, COVID IC and Historical non-IC groups, respectively. Lastly, the median total cost was $12,232, $14,984 and $11,476 for Pre-COVID IC, COVID IC and Historical non-IC groups, respectively. Although the Historical non-IC group had a lower median cost, this group tended to have greater variation in costs relative to the other groups, indicated by the larger SD and range. Similar patterns were observed with costs summarized by direct and indirect functions (Table A4).

After adjustment, the estimated RMD for the Pre-COVID IC group in the low care path indicated a lower, albeit not statistically significant, mean index cost relative to the Historical non-IC group in the low care path (0.76 (0.58–1.00)). Similarly, the estimated RMD for the COVID IC group in the low care path indicated a lower, albeit not statistically significant, mean index cost (0.96 (0.77–1.20)).

After adjustment, the estimated RMD for the Pre-COVID IC group in the medium care path indicated a statistically significantly lower mean index cost relative to the Historical non-IC group in the medium care path (0.72 (0.52–0.99)). The estimated RMD for the COVID IC group in the medium care path also indicated a lower, albeit not statistically significant, mean index cost (0.80 (0.62–1.03)).

After adjustment, the estimated RMD for the Pre-COVID IC group in the high care path indicated a higher, albeit not statistically significant, mean index cost relative to the Historical non-IC group in the high care path (1.23 (0.80–1.90)). Similarly, the estimated RMD for the COVID IC group in the high care path indicated a higher, albeit not statistically significant, mean index cost (1.38 (0.97–1.95)) (Table 4).

The results observed for total costs in the low, medium and high care paths was similar in the IC groups, compared to the historical non-IC group (Table 4).

### Wait times

Overall, the Pre-COVID IC cohort had a longer median wait time (days) from referral to first clinical appointment compared to the other groups (median (IQR) for Pre-COVID IC: 10 (5–18); COVID IC: 7 (1–15); Historical non-IC: 9 (3–17)). The Historical non-IC cohort had a longer median wait time from decision to surgery (17 (9–28); 22 (13–39); 23 (10–37)) (Table A5).

When stratified by care path, we observed that the Pre-COVID IC group tended to have a longer and more variable wait time from referral to first clinical appointment compared to the other groups across all care paths, while the COVID IC and Historical non-IC groups tended to have a longer median and more variable wait time from decision to surgery compared to the Pre-COVID IC group (Table A5).

### Home care

Among the 612 (out of 869) COVID-IC patients who were admitted to the home care program and had home care data available, nearly all patients received a planned IC Lead call (95.8%), while 17.3% of patients received additional unplanned IC Lead calls. Just under half (47.4%) received in-person nursing services, 66.3% received nursing call services, and only a few patients (0.5%) received virtual nursing services. Utilization of allied health professional services was moderate, with 10.9% receiving in-person services and 18.0% receiving virtual services. In-person personal support worker services were minimal, with only 2.5% of patients receiving these services. Lastly, the median (IQR) home care costs were CAD $48 ($20-$292), with these costs heavily skewed to the right with most patients having $0 costs, and the maximum cost was $12,779 (Table A7).

### Sensitivity analyses

Results from sensitivity analyses are reported in the Appendix.

## Discussion

Our findings suggest that the IC Program was associated with statistically significant improvements in several outcomes. Specifically, for patients in the low care path, the estimated RRs in the COVID IC group indicated a statistically significantly lower 90-day readmission risk and in the Pre-COVID IC group indicated a statistically significantly lower 90-day ED visit risk (relative to the Historical non-IC group). The estimated RMDs in both the Pre-COVID and COVID IC groups in the low care path indicated a statistically significantly shorter mean index LOS. For patients in the medium care path, the estimated RMDs in both Pre-COVID IC and COVID IC groups indicated a statistically significantly shorter mean index LOS, and the RMD in the Pre-COVID IC group indicated a statistically significantly lower mean index cost. Within the high care path, results were mixed, with a statistically significantly longer mean index LOS for the Pre-COVID IC group.

A process evaluation of the IC Program was previously conducted where patients and caregivers relayed the importance of contact with an IC Lead and availability of home care services. These contacts may have provided patients with confidence, appropriate information, and reassurance that their symptoms did not necessitate a visit to the ED [23]. These results align with the current study’s findings of a statistically significantly lower 90-day ED visit risk among the Pre-COVID IC patients in the low care path.

Furthermore, our findings, which also demonstrated a statistically significantly lower 90-day ED visit risk in the Pre-COVID low care path, shorter LOS in the low and medium care paths for both Pre-COVID and COVID IC groups, and lower mean index cost in the Pre-COVID medium care path, show similarities to those of the ICC program evaluation at SJHH [16]. ICC Patients at SJHH who underwent partial VATS procedures experienced shorter median LOS compared to historical non-ICC patients who received the same procedures, similar to the results for the UHN IC patients in the low care path. Despite also reporting a shorter adjusted mean LOS for IC patients in the medium care path that is comparable to the reduced index hospitalization LOS that was found for SJHH ICC patients who received open partial procedures, we found a statistically significantly longer adjusted mean index LOS for Pre-COVID IC patients in the high care path relative to the Historical non-IC patients. An updated SJHH ICC program evaluation was conducted for the 2010 to 2014 period and reported similar results to the initial evaluation, except for the ICC group having a statistically significantly lower risk of 60-day readmissions compared to the historical control group [18]. Although our estimates suggested a lower risk of 60-day readmissions for the IC groups relative to the Historical non-IC group, we lacked statistical precision to conclude statistical significance.

Our findings indicate a statistically significantly lower mean index cost for Pre-COVID IC patients in the medium care path, though other observed trends toward a lower mean index cost in the low and medium care paths did not reach statistical significance and warrant further analysis with larger sample sizes. At SJHH, mean costs were lower for patients who underwent partial and total VATS procedures in the initial ICC program evaluation, which the authors attributed to shorter LOS, and fewer ED visits and readmissions in the ICC cohort [16, 18]. An evaluation of the Enhanced Recovery (ER) pathway, which is an IC model for thoracic surgery patients in the UK that combines perioperative interventions, found cost-savings alongside reduced readmissions and shorter postoperative LOS comparing IC patients to historical controls [17]. We report similar results for the Pre-COVID IC group in the medium care path, but a statistically signicantly higher mean index cost for the Pre-COVID IC group in the high care path. However, the estimates were slightly less pronounced when considering total costs, rather than only index costs, particularly for the high care path, suggesting these results may be driven by costs incurred during the index surgical visit. A possible explanation for these results may be increases in healthcare costs over time due to changes in clinical practice, advancements in technology and health-sector price inflation that tends to outpace general inflation and may be most noticeable among surgical approaches used for the most complex patients in the high care path [40].

The key strengths of our evaluation are the comprehensive set of patient outcomes considered, the large sample sizes of our IC and Historical non-IC groups, and the inclusion two distinct time periods of clinical relevance (i.e., COVID IC group), which is especially important for understanding IC patient outcomes in context of the COVID-19 pandemic. However, there are limitations to our evaluation. Despite our large sample sizes, all surgeries took place within the UHN, and thus, our results are limited to a single network of hospitals and future studies should consider including multiple hospital networks. We may not eliminate all sources of bias using the historical control group due to pre-existing trends in the outcomes due to improvements in clinical practice and temporal variation in the prognosis of surgical patients [41, 42]; however, we mitigated this by adjusting for patient characteristics. We also cannot rule out any secular trends with changes in clinical care practices, such as improvement in surgical procedures that may explain some of the notable results observed for the IC groups relative to the Historical non-IC group, or more generally, non-contemporaneous bias, that may be influencing our results mostly away from the null. We were also unable to adjust for disease severity, or more generally, factors related to patient acuity and treatment modality due to data limitations. Although we stratified by care path, there is likely further necessary adjustment to minimize confounding due to disease severity and frailty. We also acknowledge the limitation of using surgical procedure alone for care path assignment in the historical control group. Due to data constraints, we were unable to incorporate comorbidities and social determinants of health into the historical group’s care path assignment, which potentially introduced imbalances in these variables between the IC and Historical non-IC groups. However, we attempted to mitigate these potential imbalances by adjusting for key demographic variables (age, sex, location of residence) and verifying acceptable comparability of primary surgical diagnoses between the groups (standardized difference < 0.3). We recommend that future research collect comprehensive data on comorbidities and social determinants of health to directly adjust for these variables to enable a more robust comparison.

Comparable post-discharge utilization and costs for home care services in the community setting were not evaluated which would have provided a more comprehensive assessment of costs. While our study aimed for a transparent evaluation of the IC Program by separating Pre-COVID and COVID periods, a limitation is the considerable difference in the length of observation for the COVID IC group (31 months) compared to the Pre-COVID IC and Historical non-IC groups (9 months each). This imbalance in length of observation and group size, largely driven by data availability, should be considered when generalizing findings, particularly from the longer COVID IC period. Lastly, the reported patient outcomes and costs for the COVID IC group are likely to be affected by the COVID-19 pandemic that began in March 2020. Specifically, the volumes of ED visits were likely underestimated due to avoidance of EDs for less urgent visits, changes to where patients sought healthcare services, and the implementation of public health measures. Indeed, ED visits in Canada significantly decreased from 15.1 million in 2019–2020 to 11.7 million in 2020–2021 [43]. Moreover, readmission rates, LOS, and costs likelywere impacted by COVID-19 [44]. With the data available in this evaluation, the analyses were not able to directly examine the impacts of COVID-19 on health outcomes and costs for IC patients, which represents an area that should be examined in future research.

## Conclusion

In this evaluation, we found that the Thoracic Surgery IC Program was associated with statistically significantly lower 90-day risk for readmission among the COVID IC patients and for ED visits among Pre-COVID IC patients in the low care path, and shorter mean index LOS for Pre-COVID and COVID IC patients in both the low and medium care paths. A statistically significantly lower mean index cost was also observed for Pre-COVID IC patients in the medium care path. The results were mixed and highly variable in the high care path, including a statistically significantly longer mean index LOS for Pre-COVID IC group, likely due to the small sample size and unmeasured factors related to the complex needs of these patients. Overall, these findings suggest that IC may help improve postoperative healthcare utilization among patients undergoing thoracic surgery.

## Supplementary Information

Supplementary Material 1.

## Data Availability

The datasets analysed for the current study are not publicly available as they are protected health data; the information is personal or special category personal data, and there is risk of “re-identification” of data that have been pseudonymised. Access to protected health data is subject to robust governance protocols, where it is lawful, ethical and safe to do. Access to protected health data is always strictly controlled using legally binding data-sharing contracts.
