# Supplementary material for: Evaluation of an integrated care program for thoracic surgery in Ontario, Canada: a historical cohort study
Source: BMC Health Serv Res. 2025 Aug 7;25:1039. doi: 10.1186/s12913-025-13049-1 (PMC12333262; doi:10.1186/s12913-025-13049-1)
Supplement: Supplementary file 1 — Supplementary Material 1. [file 12913_2025_13049_MOESM1_ESM.docx]

**SUPPLEMENTARY INFORMATION FILES: APPENDIX**

**Title:** Evaluation of an Integrated Care Program for Thoracic Surgery in Ontario, Canada:

A historical cohort study

Nicholas Bakewell^1^, Catherine Liang^1^, Tsoleen Ayanian^2^, Sanjana Sundaram^2^, Kazuhiro Yasufuku^2^, Meghan O’Neill^1^, Kathy Kornas^1^, Lori Diemert^1^, Megan Samantha Lowe^1^, Melissa Chang^2^, Laura C. Rosella^1,3^

^1^Dalla Lana School of Public Health, University of Toronto, Toronto, Ontario, CA.

^2^University Health Network, Toronto, Ontario, CA.

^3^Temerty Faculty of Medicine, University of Toronto, Toronto, Ontario, CA.

**Calculation of healthcare costs**

The following outlines how healthcare costs were calculated in the University Health Network Case Costing database.

- **Direct costs**: Costs from patient care functional centers (FCs; i.e., where costs are “charged” to) such as nursing, intensive care unit, operating room, laboratory, and pharmacy and allied health.
  - **Variable direct costs**: Costs from patient care FCs and only including Unit Producing Personnel (UPP) compensation and medical/surgical supplies, which would expect to change due to volume.
  - **Fixed direct costs:** Costs from patient care FCs and only including non-UPP compensation, sundry expense, and equipment expense, which would expect not to change due to volume.
- **Indirect costs:** Costs from supportive overhead FCs such as general administration, human resource, finance, decision support, plant operation, and housekeeping.
  - **Variable indirect costs:** Costs from overhead FCs and only including UPP compensation and supplies, which would expect to change due to volume.

**Fixed indirect costs:** Costs from overhead FCs and only including non-UPP compensation and equipment expense, which would expect not to change due to volume.

- **Total costs** were calculated as the sum of direct and indirect costs.
- The following costs were not included in the calculation of direct and indirect costs: physician costs are paid by the Ontario Health Insurance Plan revenue, and are not considered as hospital cost.

Table A1. 90-day Readmissions, 90-day ED Visits and Length of Stay among Integrated Care (IC) and Historical groups accounting for data outside of University Health Network sites, observed

|  | Pre-COVID IC | | | | COVID IC | | | | Historical non-IC | | | |
| --- | --- | --- | --- | --- | --- | --- | --- | --- | --- | --- | --- | --- |
|  | **Denominator/N** | **n (%)/ Mean (SD)** | **Median (IQR)** | **Min- Max** | **Denominator/N** | **n (%)/ Mean (SD** | **Median (IQR)** | **Min- Max** | **Denominator/N** | **n (%)/ Mean (SD)** | **Median (IQR)** | **Min- Max** |
| 90-day Readmissions | 269 | 25 (9.3%) | 1 (1, 1) | 1-2 | 869 | 88 (10.1%) | 1 (1, 1) | 1-3 | 434 | 65 (15.0%) | 1 (1, 1) | 1-3 |
| 90-day ED visits | 269 | 52 (19.3%) | 1 (1, 2) | 1-5 | 869 | 200 (23.0%) | 1 (1, 2) | 1-5 | 434 | 122 (28.1%) | 1 (1, 2) | 1-6 |
| Total  (Index + Readmission) | 269 | 6 (9) | 4 (2, 7) | 1-82 | 869 | 5 (7) | 3 (2, 6) | 1-70 | 434 | 7 (14) | 3 (2, 7) | 1-194 |

Notes

IC=Integrated Care, SD=Standard Deviation, IQR=Interquartile Range (Quartile 1, Quartile 3).

The count summaries are among those with readmissions/ED visits, hence why 0 is not the minimum value.

Table A2. Adjusted differences in 90-day readmissions, 90-day ED visits and Total Length of Stay for Integrated Care (IC) vs. historical groups, accounting for data outside of University Health Network sites

|  | **Care Path** | **Relative Difference (95% CI)^*^** | | **Absolute Difference (95% CI)^*^** | |
| --- | --- | --- | --- | --- | --- |
|  |  | **Pre-COVID IC** | **COVID IC** | **Pre-COVID IC** | **COVID IC** |
| **90-day Readmissions** | Low | 0.64 (0.36, 1.14) | 0.65 (0.43, 0.97) | -4.73 (-10.48, 1.03) | -4.62 ( -9.13, -0.10) |
|  | Medium | 0.82 (0.39, 1.74) | 0.59 (0.32, 1.07) | -2.90 (-14.04, 8.24) | -6.88 (-15.41, 1.65) |
|  | High | 0.15 (0.02, 1.04) | 0.80 (0.40, 1.58) | -22.42 (-38.11, -6.74) | -5.31 (-21.94, 11.32) |
| **90-day ED Visits** | Low | 0.63 (0.43, 0.92) | 0.76 (0.59, 0.99) | -9.99 (-17.52, -2.46) | -6.23 (-12.31, -0.15) |
|  | Medium | 0.87 (0.53, 1.45) | 0.79 (0.54, 1.16) | -3.82 (-17.83, 10.19) | -6.18 (-16.87, 4.50) |
|  | High | 0.43 (0.16, 1.17) | 1.03 (0.63, 1.69) | -20.41 (-41.43, 0.61) | 1.23 (-17.21, 19.67) |
| **Total LOS (Index + Readmissions)** | Low | 0.70 (0.59, 0.84) | 0.65 (0.57, 0.75) | -1.64 (-2.43, -0.85) | -1.95 (-2.59, -1.31) |
|  | Medium | 0.79 (0.59, 1.04) | 0.66 (0.54, 0.82) | -2.04 (-4.39, 0.30) | -3.14 (-4.98, -1.30) |
|  | High | 1.14 (0.83, 1.57) | 1.05 (0.82, 1.35) | 1.60 (-3.02, 6.23) | 0.81 (-2.60, 4.22) |

Notes

IC=Integrated Care, 95% CI=95% confidence interval.

Reference group=historical non-IC group.

^*^Absolute difference effect estimates are in risk-percentages (%) for readmission and ED visit outcomes, and days for length of stay outcomes.

All estimates presented from models for Total LOS, 90-day readmissions and 90-day ED visits were adjusted for age, sex, and location of residence (within vs. outside the Toronto Central local health planning region).

Total LOS was modelled using a Negative Binomial GLM (log link for relative differences, and identity link for absolute differences), and 90-day readmissions and 90-ED visits were modelling using a Poisson GLM with a log link and robust standard errors for relative differences, and Ordinary Least Squares regression with robust standard errors for absolute differences.

Table A3. Adjusted differences in Length of Stay and Case Costing Outcomes for Integrated Care (IC) vs. historical groups (Poisson GLM with robust standard errors for length of stay outcomes, and Gaussian GLM and Gamma GLM with operating costs removed for case costing outcomes)

|  | **Care Path** | **Relative Difference (95% CI)** | | **Absolute Difference (95% CI)^*^** | |
| --- | --- | --- | --- | --- | --- |
|  |  | **Pre-COVID IC** | **COVID IC** | **Pre-COVID IC** | **COVID IC** |
| **Length of Stay (LOS) Outcomes** | | | | | |
| **Sensitivity Analysis 1: Poisson Distribution** | | | | | |
| Index Hospitalization LOS | Low | 0.75 (0.52, 1.08) | 0.66 (0.48, 0.91) | -1.14 (-2.70, 0.42) | -1.50 (-2.94, -0.06) |
|  | Medium | 0.75 (0.52, 1.07) | 0.61 (0.44, 0.84) | -2.04 (-4.77, 0.69) | -3.17 (-5.66, -0.69) |
|  | High | 1.42 (0.92, 2.17) | 1.13 (0.87, 1.48) | 4.30 (-1.66, 10.25) | 1.51 (-1.65, 4.68) |
| Total LOS (Index + Readmissions) | Low | 0.75 (0.52, 1.07) | 0.68 (0.51, 0.92) | -1.39 (-3.15, 0.37) | -1.71 (-3.29, -0.13) |
|  | Medium | 0.71 (0.46, 1.09) | 0.63 (0.43, 0.91) | -2.62 (-6.33, 1.09) | -3.45 (-6.78, -0.12) |
|  | High | 1.16 (0.76, 1.79) | 1.02 (0.78, 1.33) | 1.95 (-4.30, 8.19) | 0.28 (-3.43, 3.99) |
| **Sensitivity Analysis 2: Post-Operative LOS** | | | | | |
| Postoperative LOS (Discharge date-Surgery date) | Low | 0.75 ( 0.52, 1.09) | 0.67 ( 0.48, 0.93) | -1.13 (-2.60, 0.34) | -1.39 (-2.74, -0.03) |
|  | Medium | 0.74 ( 0.52, 1.04) | 0.57 ( 0.41, 0.79) | -2.10 (-4.66, 0.46) | -3.36 (-5.76, -0.96) |
|  | High | 1.39 ( 0.88, 2.20) | 1.09 ( 0.83, 1.42) | 4.02 (-2.16, 10.20) | 0.97 (-1.99, 3.94) |
| **Case Costing Outcomes** | | | | | |
| **Sensitivity Analysis 1: Gaussian Distribution** | | | | | |
| Index Visit Costs | Low | 0.76 (0.55, 1.05) | 0.96 (0.76, 1.20) | -3620.11 (-7749.61, 509.38) | -657.36 (-4035.17, 2720.46) |
|  | Medium | 0.70 (0.48, 1.01) | 0.78 (0.60, 1.01) | -7124.53 (-14442.19, 193.14) | -5072.30 (-10825.37, 680.77) |
|  | High | 1.36 (0.81, 2.28) | 1.50 (0.98, 2.31) | 10879.07 (-10493.74, 32251.88) | 15760.00 (-1650.91, 33170.92) |
| Total Costs (Index + Post-discharge costs) | Low | 0.81 (0.59, 1.09) | 1.00 (0.80, 1.24) | -3193.95 (-7438.33, 1050.42) | -83.18 (-3554.97, 3388.61) |
|  | Medium | 0.66 (0.41, 1.05) | 0.76 (0.56, 1.04) | -9054.79 (-19036.09, 926.51) | -6101.90 (-13949.09, 1745.29) |
|  | High | 1.24 (0.75, 2.05) | 1.45 (0.97, 2.17) | 7611.27 (-14196.83, 29419.37) | 15142.94 (-2622.57, 32908.45) |
| **Sensitivity Analysis 2: Operating Costs Excluded** | | | | | |
| Index Visit Costs | Low | 0.70 (0.48, 1.03) | 0.79 (0.57, 1.09) | -3068.97 (-6408.82, 270.89) | -2088.77 (-5097.09, 919.55) |
|  | Medium | 0.66 (0.44, 0.99) | 0.72 (0.52, 0.99) | -6122.24 (-12171.26, -73.23) | -5039.05 (-10362.24, 284.14) |
|  | High | 1.30 (0.77, 2.21) | 1.43 (0.93, 2.19) | 5052.98 (-10861.94, 20967.90) | 10347.30 (-3255.28, 23949.88) |
| Total Costs (Index + Post-discharge costs) | Low | 0.74 (0.50, 1.09) | 0.84 (0.61, 1.16) | -2934.85 (-6526.11, 656.42) | -1700.52 (-4934.80, 1533.75) |
|  | Medium | 0.64 (0.41, 1.00) | 0.73 (0.51, 1.04) | -7183.14 (-14705.35, 339.08) | -5366.02 (-12061.68, 1329.65) |
|  | High | 1.17 (0.70, 1.94) | 1.35 (0.89, 2.04) | 1902.09 (-14035.17, 17839.35) | 9655.52 (-4625.86, 23936.91) |
| **Sensitivity Analysis 3: Operating Costs Excluded, Variable Direct Costs Only** | | | | | |
| Index Visit Costs | Low | 0.68 (0.45, 1.03) | 0.71 (0.51, 1.00) | -2031.18 (-4244.49, 182.13) | -1813.79 (-3773.61, 146.02) |
|  | Medium | 0.65 (0.42, 0.99) | 0.66 (0.47, 0.92) | -4076.80 (-8087.54, -66.07) | -3944.03 (-7439.76, -448.30) |
|  | High | 1.29 (0.75, 2.22) | 1.34 (0.86, 2.08) | 3068.80 (-7522.41, 13660.00) | 5198.92 (-3600.84, 13998.69) |
| Total Costs (Index + Post-discharge costs) | Low | 0.72 (0.48, 1.09) | 0.76 (0.54, 1.07) | -1957.43 (-4338.18, 423.32) | -1631.04 (-3734.13, 472.05) |
|  | Medium | 0.63 (0.39, 1.00) | 0.67 (0.46, 0.97) | -4731.09 (-9683.19, 221.01) | -4175.44 (-8539.36, 188.47) |
|  | High | 1.15 (0.68, 1.94) | 1.26 (0.82, 1.93) | 1013.41 (-9591.92, 11618.75) | 4626.52 (-4619.89, 13872.92) |

Notes

IC=Integrated Care, 95% CI=95% confidence interval.

Reference group=historical non-IC group.

^*^Absolute difference effect estimates are in days for length of stay outcomes, and Canadian dollars ($) for cost outcomes.

All estimates presented from models for LOS and case costing outcomes were adjusted for age, sex, and location of residence (within vs. outside the Toronto Central local health planning region).

Table A4. Length of Stay and Case Costing Outcomes among Integrated Care (IC) and Historical non-IC groups, observed

|  | Pre-COVID IC | | | | COVID IC | | | | Historical non-IC | | | |
| --- | --- | --- | --- | --- | --- | --- | --- | --- | --- | --- | --- | --- |
|  | **Denominator/N** | **Mean (SD)** | **Median (IQR)** | **Min- Max** | **Denominator/N** | **Mean (SD)** | **Median (IQR)** | **Min- Max** | **Denominator/N** | **Mean (SD)** | **Median (IQR)** | **Min- Max** |
| Length of Stay Outcomes (days) | | | | | | | | | | | | |
| Index visit | 269 | 5 (7) | 3 (2, 6) | 1-82 | 869 | 5 (5) | 3 (2, 6) | 1-70 | 434 | 6 (12) | 3 (2, 6) | 1-194 |
| Readmissions 90 days post- discharge | 25 | 6 (7) | 3 (2, 8) | 1-28 | 85 | 8 (11) | 4 (2, 9) | 1-64 | 63 | 7 (9) | 5 (2, 9) | 1-54 |
| Total (Index + Readmissions) | 269 | 6 (8) | 3 (2, 7) | 1-82 | 869 | 5 (7) | 3 (2, 6) | 1-70 | 434 | 7 (14) | 3 (2, 7) | 1-194 |
| Case Costing Outcomes (Canadian dollars (CAD$)) | | | | | | | | | | | | |
| Index Visit | | | | | | | | | | | | |
| Total | 267 | 16438 (21910) | 11472 (8224, 17524) | 4001-318498 | 671 | 19278 (19929) | 14285 (10263, 20530) | 3888-276227 | 376 | 19464 (36748) | 10903 (6861, 19945) | 1919-546277 |
| Direct | 267 | 11888 (15988) | 8264 (5929, 12796) | 2769-232474 | 671 | 13291 (13993) | 9826 (7034, 14123) | 2730-195799 | 376 | 14211 (26875) | 7938 (4988, 14585) | 1302-399389 |
| Indirect | 267 | 4550 (5930) | 3197 (2298, 4791) | 1213-86024 | 671 | 5987 (5964) | 4556 (3193, 6419) | 1158-80428 | 376 | 5253 (9885) | 2977 (1932, 5357) | 616-146888 |
| Readmission and/or ED visits 90-days post-index discharge | | | | | | | | | | | | |
| Total | 38 | 7784 (10127) | 3881 (640, 10914) | 254-39155 | 111 | 9867 (14957) | 4028 (1297, 11826) | 58-103966 | 80 | 7504 (20648) | 2437 (387, 5931) | 92-170786 |
| Direct | 38 | 5521 (7203) | 2733 (452, 7650) | 176-28328 | 111 | 6756 (10688) | 2672 (855, 8040) | 40-79559 | 80 | 5412 (14905) | 1732 (279, 4210) | 64-122614 |
| Indirect | 38 | 2263 (2938) | 1148 (191, 3194) | 78-10863 | 111 | 3111 (4362) | 1336 (439, 3786) | 19-24407 | 80 | 2092 (5754) | 698 (111, 1716) | 28-48172 |
| Overall (Index+ Post-index visits) | | | | | | | | | | | | |
| Total | 267 | 17546 (22803) | 12232 (8610, 18527) | 4001-318498 | 671 | 20910 (21716) | 14984 (10561, 21941) | 3888-288160 | 376 | 21060 (41616) | 11476 (7334, 21363) | 1919-546277 |
| Direct | 267 | 12674 (16611) | 8820 (6215, 13333) | 2769-232474 | 671 | 14409 (15238) | 10357 (7312, 15099) | 2730-203988 | 376 | 15363 (30370) | 8410 (5344, 15793) | 1302-399389 |
| Indirect | 267 | 4872 (6201) | 3413 (2395, 5151) | 1232-86024 | 671 | 6502 (6515) | 4704 (3267, 6872) | 1158-84172 | 376 | 5698 (11259) | 3095 (1989, 5692) | 616-146888 |

Notes

IC=Integrated Care, SD=Standard Deviation, IQR=Interquartile Range (Quartile 1, Quartile 3).

Healthcare cost data not available for all patients.

Table A5. Summary of surgical wait times among Integrated Care (IC) and Historical non-IC groups, observed

| ***Unit: days*** | **Pre-COVID IC** | | | **COVID IC** | | | **Historical non-IC** | | |
| --- | --- | --- | --- | --- | --- | --- | --- | --- | --- |
|  | Mean (SD) | Median (IQR) | Min-Max | Mean (SD) | Median (IQR) | Min-Max | Mean (SD) | Median (IQR) | Min-Max |
| **Overall^*^:** | N=205/269 | | | N=694/869 | | | N=280/434 | | |
| Time from first referral to first clinical appointment (Wait 1) | 15 (24) | 10 (5, 18) | 0-230 | 13 (22) | 7 (1, 15) | 0-339 | 13 (17) | 9 (3, 17) | 0-175 |
| Time from decision to surgery (Wait 2) | 21 (21) | 17 (9, 28) | 0-176 | 35 (45) | 22 (13, 39) | 0-451 | 32 (38) | 23 (10, 37) | 0-327 |
| **Low care path:** | N=139/177 | | | N=445/520 | | | N=197/300 | | |
| Time from first referral to first clinical appointment (Wait 1) | 16 (25) | 10 (5, 16) | 0-230 | 12 (21) | 8 (1, 15) | 0-339 | 13 (17) | 10 (3, 17) | 0-175 |
| Time from decision to surgery (Wait 2) | 20 (19) | 16 (9, 29) | 0-176 | 30 (35) | 21 (13, 36) | 0-451 | 31 (35) | 23 (10, 38) | 0-327 |
| **Medium care path:** | N=46/65 | | | N=175/243 | | | N=58/96 | | |
| Time from first referral to first clinical appointment (Wait 1) | 16 (27) | 9 (4, 19) | 0-154 | 15 (24) | 7 (0, 17) | 0-130 | 15 (21) | 7 (0, 18) | 0-97 |
| Time from decision to surgery (Wait 2) | 26 (29) | 19 (10, 29) | 0-149 | 45 (56) | 27 (13, 50) | 0-407 | 39 (53) | 23 (10, 37) | 0-244 |
| **High care path:** | N=19/26 | | | N=54/82 | | | N=25/38 | | |
| Time from first referral to first clinical appointment (Wait 1) | 12 (10) | 10 (4, 19) | 0-31 | 14 (19) | 8 (3, 15) | 0-83 | 10 (7) | 9 (3, 14) | 0-27 |
| Time from decision to surgery (Wait 2) | 17 (9) | 16 (11, 23) | 2-37 | 38 (58) | 21 (14, 29) | 6-380 | 22 (18) | 18 (12, 26) | 4-71 |

Notes

IC=Integrated Care, SD=Standard Deviation, IQR=Interquartile Range (Quartile 1, Quartile 3).

The number of patients that have data over the whole same is reported overall and by care path group.

^*^Patients that had same day procedures were classified as an “Other” care path category, and thus, were excluded from the care path summaries below the “Overall” summaries (n “Other” care path category=25).

Table A6. Adjusted differences in Readmissions, ED Visits, Length of Stay and Case Costing Outcomes for Integrated Care (IC) vs. historical groups, with adjustment for age, sex, location of residence (within vs. outside the Toronto Central local health planning region), and surgical wait time from decision to surgery

|  | **Care Path** | **Relative Difference (95% CI)** | | **Absolute Difference (95% CI)^*^** | |
| --- | --- | --- | --- | --- | --- |
|  |  | **Pre-COVID IC** | **COVID IC** | **Pre-COVID IC** | **COVID IC** |
| **Readmissions** | | | | | |
| 90 days | Low | 0.68 (0.32, 1.44) | 0.71 (0.42, 1.20) | -3.78 (-10.03, 2.46) | -2.90 (-7.72, 1.92) |
|  | Medium | 1.06 (0.41, 2.75) | 0.71 (0.31, 1.62) | 0.36 (-13.63, 14.35) | -3.58 (-14.18, 7.02) |
|  | High | 0.23 (0.03, 1.93) | 1.03 (0.41, 2.58) | -17.97 (-38.95, 3.02) | 0.54 (-21.72, 22.80) |
| 60 days | Low | 0.74 (0.32, 1.73) | 0.73 (0.40, 1.34) | -2.36 (-7.87, 3.16) | -2.02 (-6.25, 2.21) |
|  | Medium | 1.17 (0.36, 3.81) | 0.85 (0.30, 2.44) | 0.96 (-10.79, 12.71) | -0.65 (-9.22, 7.91) |
|  | High | 0.26 (0.03, 2.31) | 1.15 (0.42, 3.13) | -14.67 (-34.53, 5.19) | 2.06 (-19.09, 23.22) |
| 30 days | Low | 0.75 (0.28, 2.05) | 0.87 (0.43, 1.75) | -1.74 (-6.61, 3.12) | -0.68 (-4.41, 3.06) |
|  | Medium | 1.56 (0.37, 6.61) | 1.10 (0.27, 4.41) | 2.55 (-7.54, 12.65) | 0.98 (-6.35, 8.31) |
|  | High | 0.42 (0.04, 4.17) | 1.63 (0.47, 5.64) | -6.65 (-23.11, 9.80) | 6.91 (-10.28, 24.10) |
| **ED Visits** | | | | | |
| 90 days | Low | 0.54 (0.25, 1.14) | 0.87 (0.54, 1.39) | -9.16 (-16.50, -1.82) | -4.25 (-10.37, 1.87) |
|  | Medium | 0.79 (0.24, 2.68) | 0.72 (0.28, 1.89) | -8.43 (-23.13, 6.27) | -8.40 (-20.32, 3.53) |
|  | High | 0.45 (0.09, 2.13) | 1.03 (0.41, 2.55) | -14.31 (-42.22, 13.61) | -1.11 (-25.50, 23.28) |
| 60 days | Low | 0.57 (0.30, 1.08) | 0.76 (0.50, 1.16) | -7.04 (-14.04, -0.05) | -3.53 (-9.27, 2.21) |
|  | Medium | 0.59 (0.21, 1.64) | 0.59 (0.29, 1.21) | -7.79 (-21.34, 5.76) | -6.48 (-17.29, 4.32) |
|  | High | 0.56 (0.16, 1.99) | 1.06 (0.47, 2.41) | -12.57 (-37.98, 12.84) | 2.12 (-21.49, 25.73) |
| 30 days | Low | 0.54 (0.25, 1.14) | 0.87 (0.54, 1.39) | -6.02 (-12.26, 0.22) | -1.53 (-6.80, 3.75) |
|  | Medium | 0.79 (0.24, 2.68) | 0.72 (0.28, 1.89) | -2.46 (-13.96, 9.04) | -2.49 (-11.69, 6.71) |
|  | High | 0.45 (0.09, 2.13) | 1.03 (0.41, 2.55) | -13.10 (-36.18, 9.97) | 0.51 (-21.60, 22.62) |
| **Length of Stay (LOS) Outcomes** | | | | | |
| Index Hospitalization LOS | Low | 0.90 (0.76, 1.07) | 0.84 (0.73, 0.95) | -0.43 (-0.98, 0.12) | -0.59 (-1.01, -0.16) |
|  | Medium | 0.79 (0.60, 1.03) | 0.58 (0.47, 0.72) | -1.40 (-3.30, 0.49) | -3.21 (-4.66, -1.75) |
|  | High | 1.71 (1.21, 2.41) | 1.12 (0.84, 1.48) | 6.42 (1.86, 10.98) | 1.36 (-1.47, 4.19) |
| Total LOS (Index + Readmissions) | Low | 0.86 (0.72, 1.04) | 0.87 (0.76, 1.01) | -0.53 (-1.20, 0.13) | -0.52 (-1.03, 0.00) |
|  | Medium | 0.81 (0.60, 1.10) | 0.61 (0.48, 0.77) | -1.33 (-3.86, 1.19) | -3.47 (-5.37, -1.56) |
|  | High | 1.34 (0.93, 1.93) | 1.01 (0.75, 1.35) | 3.85 (-1.43, 9.12) | 0.39 (-3.33, 4.12) |
| **Case Costing Outcomes** | | | | | |
| Index Visit Costs | Low | 0.92 (0.79, 1.07) | 1.16 (1.03, 1.31) | -1181.40 (-2983.91, 621.10) | 2066.27 (475.21, 3657.33) |
|  | Medium | 0.76 (0.55, 1.05) | 0.83 (0.64, 1.08) | -4592.77 (-11148.37, 1962.83) | -3216.51 (-8708.02, 2274.99) |
|  | High | 1.40 (0.87, 2.27) | 1.17 (0.78, 1.74) | 9962.74 (-9498.33, 29423.80) | 4655.54 (-9427.83, 18738.90) |
| Total Costs (Index + Post-discharge costs) | Low | 0.93 (0.80, 1.09) | 1.19 (1.05, 1.35) | -1113.75 (-3111.57, 884.08) | 2518.28 (751.02, 4285.54) |
|  | Medium | 0.77 (0.54, 1.10) | 0.84 (0.63, 1.12) | -4779.24 (-12685.14, 3126.67) | -3284.63 (-9813.66, 3244.40) |
|  | High | 1.30 (0.81, 2.09) | 1.14 (0.77, 1.70) | 9428.85 (-10406.56, 29264.26) | 6834.59 (-8694.33, 22363.51) |

Notes

IC=Integrated Care, 95% CI=95% confidence interval.

Reference group=historical non-IC group.

^*^Absolute difference effect estimates are in risk-percentages (%) for readmission and ED visit outcomes, days for length of stay outcomes, and Canadian dollars ($) for cost outcomes.

Sample sizes of each care path: low (N=781), medium (N=279), high (N=98) for readmissions, ED visits and LOS outcomes; low (N=679), medium (N=240), high (N=82) for healthcare cost outcomes.

Table A7. Summary of home care utilization among Integrated Care patients enrolled in home care and with data available

|  | **n (%)** | **Mean (SD)** | **Median (IQR)** | **Min-Max** |
| --- | --- | --- | --- | --- |
| **UHN IC Lead Call** |  |  |  |  |
| Planned* | 586 (95.8%) | 2.22 (0.92) | 2 (2, 3) | 0-8 |
| Unplanned | 106 (17.3%) | 0.27 (0.71) | 0 (0, 0) | 0-7 |
| **Nursing** |  |  |  |  |
| In-person | 290 (47.4%) | 1.78 (6.07) | 0 (0, 2) | 0-100 |
| Virtual | 3 (0.5%) | 0.00 (0.07) | 0 (0, 0) | 0-1 |
| Call* | 406 (66.3%) | 1.17 (1.08) | 1 (0, 2) | 0-5 |
| **Allied Health Professionals** |  |  |  |  |
| In-person | 67 (10.9%) | 0.19 (0.69) | 0 (0, 0) | 0-8 |
| Virtual | 110 (18.0%) | 0.61 (1.64) | 0 (0, 0) | 0-10 |
| **Personal Support Workers, in-person** | 15 (2.5%) | 0.12 (0.94) | 0 (0, 0) | 0-13 |
| **Costs (Canadian dollars (CAD$))** |  | 262.07 (785.82) | 48.48 (20.00, 291.94) | 0.00-12778.81 |
| Services |  | 2.64 (23.16) | 0.00 (0.00, 0.00) | 0.00-401.00 |
| Equipment |  | 37.24 (331.71) | 0.00 (0.00, 0.00) | 0.00-7387.76 |
| Service-related costs |  | 222.19 (522.74) | 47.96 (17.72, 278.51) | 0.00-9189.09 |

Notes

SD=Standard Deviation, IQR=Interquartile Range (Quartile 1, Quartile 3).

There were 612 Integrated Care (IC) patients with home care data associated with their index thoracic surgery visit used in this evaluation, all of whom were part of the COVID IC group, as expected given that reliable data were available from November 2020 onwards.

Costs are in Canadian dollars (CAD$).

*Due to changes in data collection and reporting during the data collection period, it is possible that a planned UHN IC Lead call conducted by a nurse may have been recorded as a planned nursing call.
